# Spontaneous Cannabinoid Receptor 2 (CB_2_) Expression in the Cochlea of Adult Albino Rat and Its Up-Regulation after Cisplatin Treatment

**DOI:** 10.1371/journal.pone.0161954

**Published:** 2016-08-26

**Authors:** Sergio Martín-Saldaña, Almudena Trinidad, Elvira Ramil, Antonio J. Sánchez-López, Maria José Coronado, Esther Martínez-Martínez, José Miguel García, José Ramón García-Berrocal, Rafael Ramírez-Camacho

**Affiliations:** 1 Ear Research Group, Universitary Hospital Puerta de Hierro Majadahonda- Health Research Institute Puerta de Hierro, Madrid, Spain; 2 Sequencing and Molecular Biology Unit, Universitary Hospital Puerta de Hierro Majadahonda, Health Research Institute Puerta de Hierro, Madrid, Spain; 3 Neuroimmunology Unit, Universitary Hospital Puerta de Hierro Majadahonda- Health Research Institute Puerta de Hierro, Madrid, Spain; 4 Confocal Microscopy Unit, Universitary Hospital Puerta de Hierro Majadahonda, Health Research Institute Puerta de Hierro, Madrid, Spain; 5 Department of Medical Oncology, Universitary Hospital Puerta de Hierro Majadahonda- Health Research Institute Puerta de Hierro, Madrid, Spain; Martin Luther University, GERMANY

## Abstract

We provide evidence for the presence of cannabinoid CB_2_ receptors in some cellular types of the cochlea of the adult albino rat. Cannabinoids and their receptors are increasingly being studied because of their high potential for clinical use. As a hyperspecialized portion of the peripheral nervous system, study of the expression and function of cannabinoid receptors in the hearing organ is of high interest. Stria vascularis and inner hair cells express CB_2_ receptor, as well as neurites and cell bodies of the spiral ganglion. Cellular types such as supporting cells and outer hair cells, in which the expression of other types of functional receptors has been reported, do not significantly express CB_2_ receptors in this study. An up-regulation of CB_2_ gene expression was detected after an ototoxic event such as cisplatin treatment, probably due to pro-inflammatory events triggered by the drug. That fact suggests promising potential of CB_2_ receptor as a therapeutic target for new treatments to palliate cisplatin-induced hearing loss and other ototoxic events which triggers inflammatory pathways.

## Introduction

There is an increasing body of research on molecular signalling in the cochlea with the expectation that a deeper understanding of cell damage and regeneration pathways could uncover potential targets for the treatment and prevention of auditory damage.

In the context of searching for potential therapeutic targets against inner ear disorders, the study of cannabinoid receptors expression in the auditory organ is of great interest. Cannabinoid receptors modulate a large number of normal brain and bodily functions and have been implicated as potential drug targets in a wide variety of diseases from cancer [1] to neurodegenerative disorders [2].

The endocannabinoid system is increasingly being studied because of the high potential for clinical use of cannabinoids. The endocannabinoid system is a cell communication system which consists of two receptors, CB_1_ and CB_2_, their endogenous ligands (anandamide and 2-AG), and the enzymes that produce and metabolize these ligands [3]. Cannabinoids exert a wide spectrum of therapeutic effects through CB_1_ and CB_2_ receptors [4]. Cannabinoid receptors are of a class of cell membrane receptors under the G protein-coupled receptor superfamily that contain seven transmembrane spanning domains [3]. Also, the existence of other cannabinoid receptors is suspected due to the actions of compounds that produce cannabinoid-like effects without activating either CB_1_ or CB_2_ [5]. CB receptors signaling pathways include regulation of adenylyl cyclase, MAP kinase, intracellular Ca ^2+^, and ion channels [6]. The first cannabinoid receptor (CB_1_) was characterized in rat brain in 1988 [7] and is mainly expressed in lung [8], liver [9] and kidneys [10]. CB_2_ was isolated in the rat spleen in 1993 [11]. CB_2_ receptor has been found to be 10–100 times more abundant than CB_1_ in immune cells such as macrophages, B cells, “natural killer” (NK) cells, monocytes, neutrophills and T cells [12]. It has also been described in non-immunological cells such as gastrointestinal system cells [13]; lung endotelium [14]; osteocytes and osteoclasts of bone [15] mouse spermatogenesis [16] and other aspects of reproduction [17]; in trabecular meshwork cells of the eye [18]; in adipocytes [19]; and in cells from cirrhotic liver (but not in the healthy organ) [20].

Cisplatin (CDDP) was the first platinum-based drug to be used and nowadays is widely employed in the treatment for some neoplastic entities such as head and neck, ovary, bladder, lung or brain. However, it presents severe side effects such as nephrotoxicity, bone marrow toxicity, gastrointestinal toxicity, liver toxicity, peripheral nervous system toxicity and ototoxicity [21]. Most of these related effects can be treated. However, CDDP- induced ototoxicity, that implies several cochlear damage which evolves in hearing impairment, tinnitus and dizzines, is one of the main reasons to chemotherapy discontinue.Inflammation has been recently shown to play a role in auditory cells apoptosis induced by CDDP, and is the main mechanism involved in immune-mediated hearing loss [22]. The inflammatory mediator inducible nitric oxide synthase (iNOS), is activated through nuclear factor-kB (NF-kB) and is related with cochlear wound by increasing cytokine expression and apoptosis in the cochlea [23]. Therefore, CDDP induce an over expression of COX-2, iNOS and TNF-α, that are regulated in cell nucleus by STAT1. STAT1 has an important role in inflammation and apoptosis in the cochlea like Kaur *et al*. demonstrated when used short interfering RNA against STAT1 to ameliorate CDDP-induced ototoxicity reducing the inflammation [24].

There is a report on the expression of CB_2_ receptors in the auditory HEI-OC1 (House Ear Institute-Organ of Corti) cell line [25]. In this study, researchers were able to demonstrate that JWH-015 (a synthetic CB_2_ agonist) could inhibit CDDP-induced apoptosis in HEI-OC1 cells.

Up to our knowledge, CB_2_ receptor has been studied *in vivo* in relation to inflammatory processes in the outer ear [26] and the middle ear [27], but there are no reports on CB_2_ receptor expression or function in the inner ear.

The aim of this study was to explore the expression of CB_2_ receptors in the mature inner ear of the Wistar rat by means of immunohistochemical staining in post-mortem tissue sections of the cochlea. CB_2_ gene expression was also measured by RT-qPCR in healthy and CDDP treated animals, using a well-established murine model of CDDP-induced ototoxicity, in order to study if an ototoxic event triggers an up- or down-transcriptional modulation of this receptor in the cochlea. These results may have important preliminary implications for the therapy of CDDP-induced hearing loss and other inflammatory inner ear diseases using CB_2_ receptor as a therapeutic target to ameliorate ototoxicity events.

## Material and Methods

### Animals

Thirty three female Wistar rats (14–16 weeks old) weighting between 200 and 280 g were used in the present study. Animals were breaded and handled at the animal facility of the Health Research Institute Puerta de Hierro in controlled temperature rooms, with light–dark cycles, and with free access to food and water. Rats with signs of present of past middle ear infection were discarded.

The animals were handled according to Spain Royal Law 53/2013 and the European Directive 2010/63/EU. The study was approved by the Clinical Research and Ethics Committee of Hospital Universitario Puerta de Hierro and the Autonomic Community of Madrid (PROEX 022/16).

### Auditory steady-state responses (ASSR)

The animals were randomly assigned in two different groups: one group (n = 20; 10 for histology and 10 for RT-PCR analysis) was administered with phosphate buffered saline (PBS) as a control, and the other group (n = 13; 3 for histology and 10 for RT-PCR) received 10 mg/kg of CDDP (Accord Healthcare, Barcelona, Spain) dose. After 72 hours animals were euthanized by CO inhalation and cochleae, spleen and kidneys of each animal were extracted. Animals were anesthetized with intraperitoneal ketamine (100 mg/kg) and diazepam (0.1 mg/kg) before the procedure.

Subcutaneous electrodes were placed over the vertex (active) and in the pinna of each ear (reference). An insert earphone (Etymotic ER-2) was placed directly into the external auditory canal. Ground electrodes were placed over the neck muscles. ASSR were recorded using an evoked potential averaging system (Intelligent Hearing System Smart-EP, FL, USA) in an electrically shielded, double-walled, sound-treated booth in response to 100 ms clicks or tone bursts, at 8, 12, 16, 20, 24, 28 and 32 kHz with 10 ms plateau and 1 ms rise/fall time. Intensity was expressed in decibels sound pressure level (dB SPL) peak equivalent. Intensity series were recorded, and an ASSR threshold was defined by the lowest intensity able to induce a replicable visual detectable response.

ASSR was measured before treatments with PBS or CDDP, and 72 hours after the administration.

### Tissue extraction, fixation and decalcification

Thirteen animals were euthanized by CO inhalation and subsequently decapitated. Temporal bones were removed and structures of middle ear dissected for isolation of the cochlear portion. Cochleae were fixed by intralabyrinthine perfusion with 4% paraformaldehyde (PFA) in phosphate buffer (pH = 7) and then immersed for 24 hours in 4% PFA solution. Afterwards, cochleae were decalcified in 0.12 M Ethylenediaminetetracetic acid dipotassium salt dihydrate (Sigma-Aldrich, St.Louis, USA) for 7–10 days. Cochleae were finally maintained in 1% PFA in 0.1 M PBS.

Spleen and kidneys of each animal were extracted and submitted to the same protocol (except the decalcification step).

### Histological processing

In order to obtain complementary images from the cellular structures of the organ of Corti, the two cochleae from one animal were each randomly processed by one of the following techniques:

#### Inner ear surface preparations

Cochleae were dissected following Liberman’s technique. In short, cochlea was bisected along a mid-modiolar plane and the half-turns cut apart, ensuring that the spiral ligament and tectorial membrane were pulled off and the resulting pieces of tissue were mounted on a microscope slide for examination in a confocal laser scanning microscope.

#### Mid-modiolar sections

The cochleae were processed for paraffin embedding. Careful placement of the cochlear portion was performed to obtain transverse serial sections of 6 μm (Leica microtome RM 2235), which were orthogonal and complementary to the surface preparations.

### Immunolabeling

CB_2_ receptors were immunostained with the polyclonal anti-cannabinoid receptor II antibody (ab45942, Abcam, USA; dilution 1:50), using Alexa 488-conjugated anti-rabbit raised in goat (Molecular Probes–Invitrogen; dilution 1:400) as the secondary antibody. Antibodies were diluted in PBS supplemented with 1% bovine serum albumin (BSA) and 0.04% triton. All preparations were contrasted with TOPRO-3 iodide (Life Technologies, USA; dilution 1:500). Specificity was confirmed in separated experiments with additional negative controls, including tissue sections incubated with primary antibody pre-adsorbed to an excess of control peptide (ab45941, Abcam, USA; dilution 1:25).

Cochleae assigned to surface preparations were immunolabeled once bisected into two halves and before final dissection, by means of a “free floating” technique. Transversal sections were immunolabeled once mounted on the slide and conveniently dried.

As a positive control of the primary antibody used, spleen and kidneys [28] of each animal were submitted to the same protocol.

### Microscopic analysis

Samples were visualized by means of a confocal laser scanning microscope Nikon Eclipse C1 coupled to a Nikon 90i microscope with a camera DXM1200F (Nikon, Haarlem, Netherlands).

For validation of the specificity of the CB_2_ immunolabelling, immunofluorescence patterns were compared to negative controls in which primary antibody was pre-adsorbed with blocking peptide, and kidney and spleen were used as a positive control.

### Measurement of immunofluorescence intensity

For relative quantification of CB_2_ fluorescence intensity, standardized settings for image acquisition and processing were used. To obtain values for CB_2_ immunofluorescence intensity in cell types of the cochlear duct, morphological boundaries of each cell type were determined on phase contrast images. The cell type-specific outlines were plotted with the corresponding grey-scaled CB_2_ immunofluorescence images. Single measurements of fluorescence intensity were performed using ImageJ software (version 1.40 g; National Institutes of Health, US). CB_2_ fluorescence intensity values for each cell type of the cochlear duct were averaged from ten independent measurements of immunolabelled cochlear mounts and sections from ten specimens. The fluorescence intensity of CB_2_ labelling was plotted using arbitrary units ranging from 0 to 2500.

Following the same protocol used to determine relative fluorescence intensity in cell types of the cochlea, effect of CDDP treatment in the immunofluorescence was compared in stria vascularis and IHC of three animals treated with CDDP and three animals treated with PBS.

### Measurement of CB_2_ gene expression by RT-qPCR in healthy and CDDP treated animals

#### Total RNA purification

Dissected cochleae obtained from CDDP-treated and control Wistar rats were embebed in RNA later overnight and kept at -80°C until purification. Tissues were resuspended in Trizol and homogenized by the MagNA Lyser System (Roche). Total RNA was isolated by RNeasy kit (QIAGEN) including on-column DNAse Digestion, following protocol previously described [29].

RNA concentration was determined by spectrophotometry and each sample was reverse-transcripted to cDNA by using the First-Strand cDNA Synthesis Protocol (Agilent Technologies).

#### Relative Quantification of CB_2_ and CB_1_ gene expression

qPCR was performed in a LC480 (Roche) in order to quantify CB_2_ and CB_1_ levels of gene expression in relation to the level of the reference TATA-binding protein (TBP), in the cochlear tissue using Real Time Ready Single Assay (TBP Transcript ID ENSRNOT0000002038, amplicon length of 75 bps from 1029 to 110; CB_2_ Transcript ID ENSRNOT00000012342, amplicon length of 65 bps from 344 to 408; CB_1_ Transcipt ID ENSRNOT00000010850, amplicon length of 66 bps from 3244 to 3309; Roche). CB_1_ gene expression was used as a control (**see S1 Fig**).

### Statistical analysis

Statistical analysis was performed with GraphPad Prism6 (San Diego, USA). One-way ANOVA was used to analyze for statistical significance of the results. Tukey test was used to identify significant differences between the paired treatments. p<0.05 was considered statistically significant.

## Results

### Ototoxic effect of CDDP-treatment

**Fig 1:**
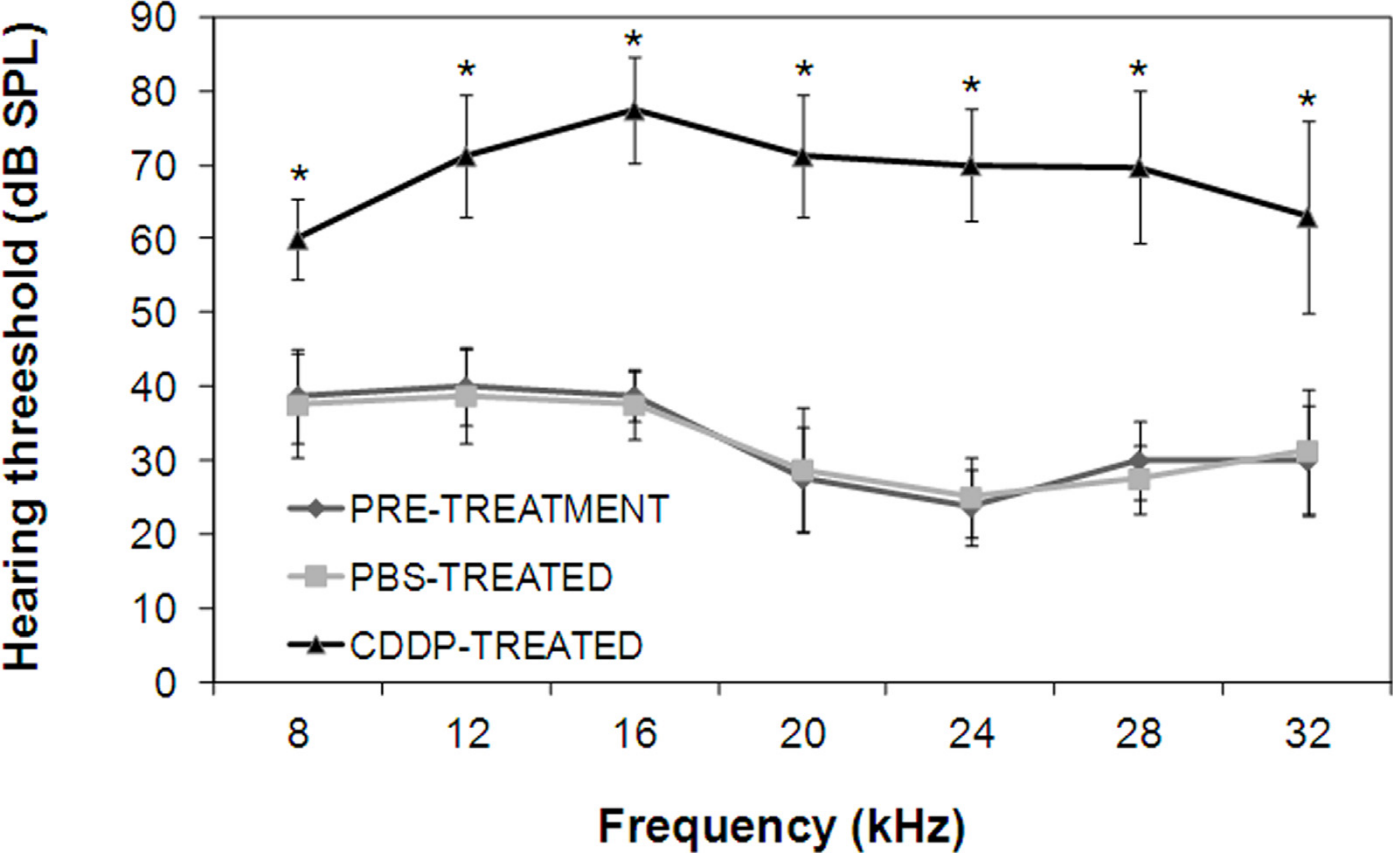
Hearing thresholds before and after intraperitoneal administration of 10 mg/kg dose of CDDP or PBS. CDDP-induced hearing loss was statistically significant for all frequencies with respect to pre-treatment hearing. The diagrams include the mean, the standard deviation (n = 33), and the ANOVA results (difference statistically significant respect tectorial membrane *p<0.05).

Post-treatment ASSR recordings were found to be higher than pre-treatment ASSR recordings in the CDDP-treated animals, as it was previously described [30]. PBS-treated animals did not suffer hearing loss (**Fig 1**).

### CB_2_ receptor detection in the cochlea by immunohistochemistry

**Fig 2:**
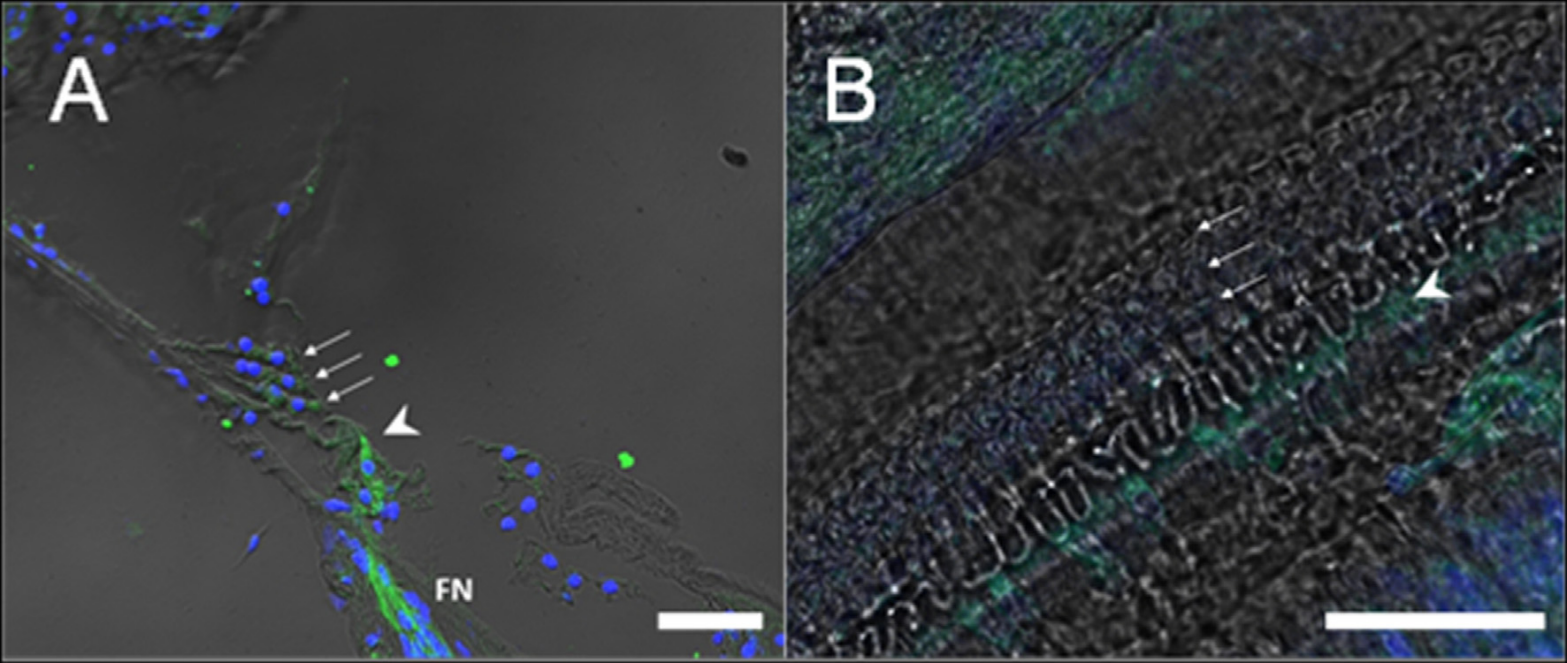
Detailed view of the sensory hair cells of the organ of Corti. Detailed view of the 3 rows of OHC (arrows) and the single row of IHC (arrowhead). (A) Mid-modiolar section. (B) Cochlear whole mount. Immunofluorescence labeling of CB_2_ receptors is shown in green. To-Pro (blue) stains nuclei of cells. Intense fluorescence is observed in IHC (arrowhead), but not in OHC. Labeling of nerve fibers can be seen at the bottom of the figure (FN). IHC: inner hair cell. OHC: outer hair cell. (Scale bar = 30μm).

CB_2_ labeling was detected in the organ of Corti, specifically in inner hair cells (IHC) (**Fig 2**).

**Fig 3:**
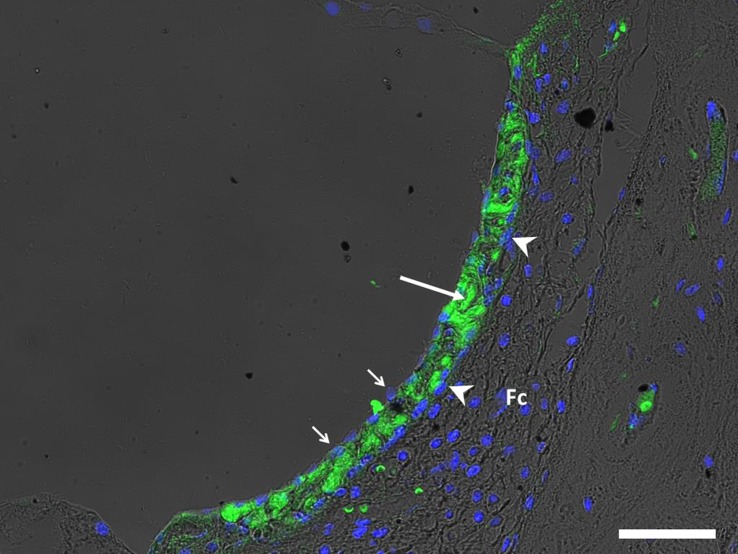
Image showing intense CB_2_ immunolabeling in cellular types of the stria vascularis (SV). Intermediate cells are indicated by long arrow. Marginal cells (short arrows); basal cells (arrowheads) might show labeling at some points. Fibrocytes of the spiral ligament (Fc) do not show expression of CB_2_ receptors. (Scale bar = 30μm)

In the stria vascularis, the CB_2_ labeling pattern was mostly coincident with an intermediate region within the stria vascularis, although some of the clearly immunolabeled cells occupied a luminal position and also the basal region in some areas. This is compatible with the distribution of intermediate cells as well as at least some of the marginal and basal cells (**Fig 3**).

**Fig 4:**
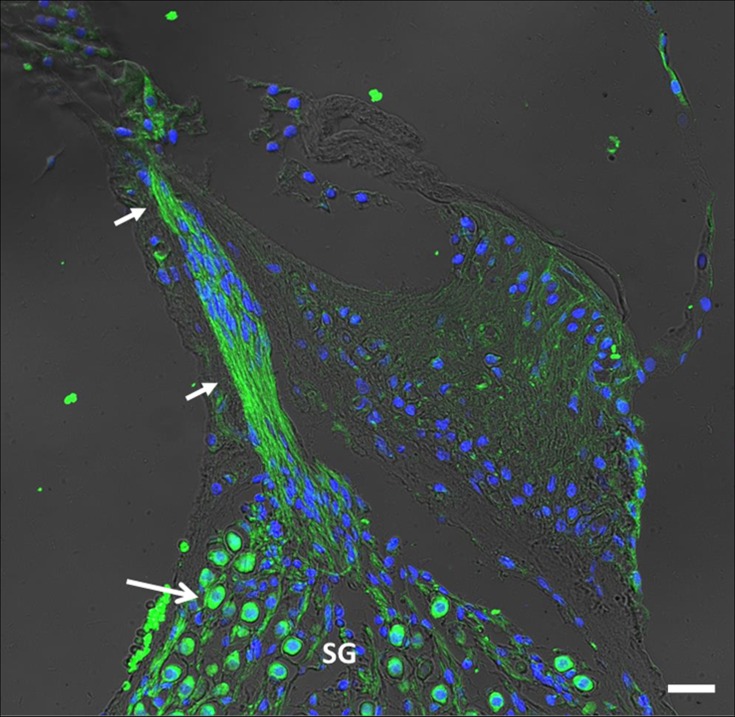
Close view of CB_2_ immunolabeling of nerve fibers and spiral ganglion. Neurites are intensely labeled (short arrows), as well as the soma (long arrow) of the spiral ganglion (SG) neurons (Scale bar = 30μm).

A strong CB_2_ labeling was also observed in the soma and neurites of the spiral ganglion neurons (**Fig 4**).

**Fig 5:**
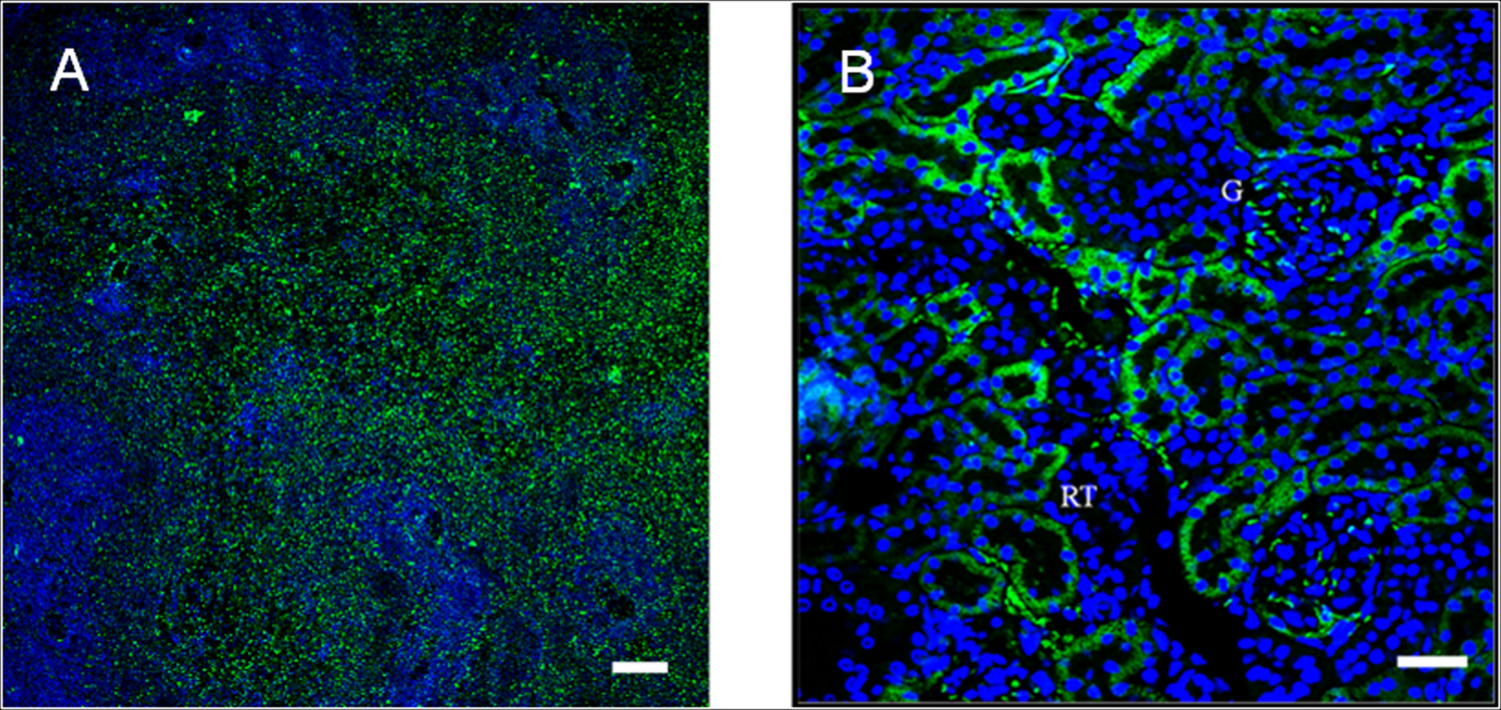
Detailed view of immunolabeling of CB_2_ in the spleen and the kidney. Detailed view of CB_2_ immunolabeling in the spleen (A), used as a positive control of the primary antibody (Scale bar = 100μm). Detailed view of CB_2_ immunolabeling of epithelial cells of the renal tubules (RT) and the glomerule (G) of the kidney (B). (Scale bar = 30μm).

As a control, the same protocol was assayed in the kidney and spleen. A strong marking was observed in the spleen related to the B cells (**Fig 5A**). A strong labeling of CB_2_ receptor in the epithelial cells of renal tubules was observed in the kidney. A slightly marking in the glomerule was also detected and the pattern of staining was suggestive of podocyte labeling, as previously described by Barutta *et al*. (**Fig 5B**). Tissue sections incubated with CB_2_ primary antibody pre-adsorbed with control peptide did not show immunofluorescence, demonstrated the specificity of primary antibody used against the CB_2_ receptor (**S1 Fig**).

**Fig 6:**
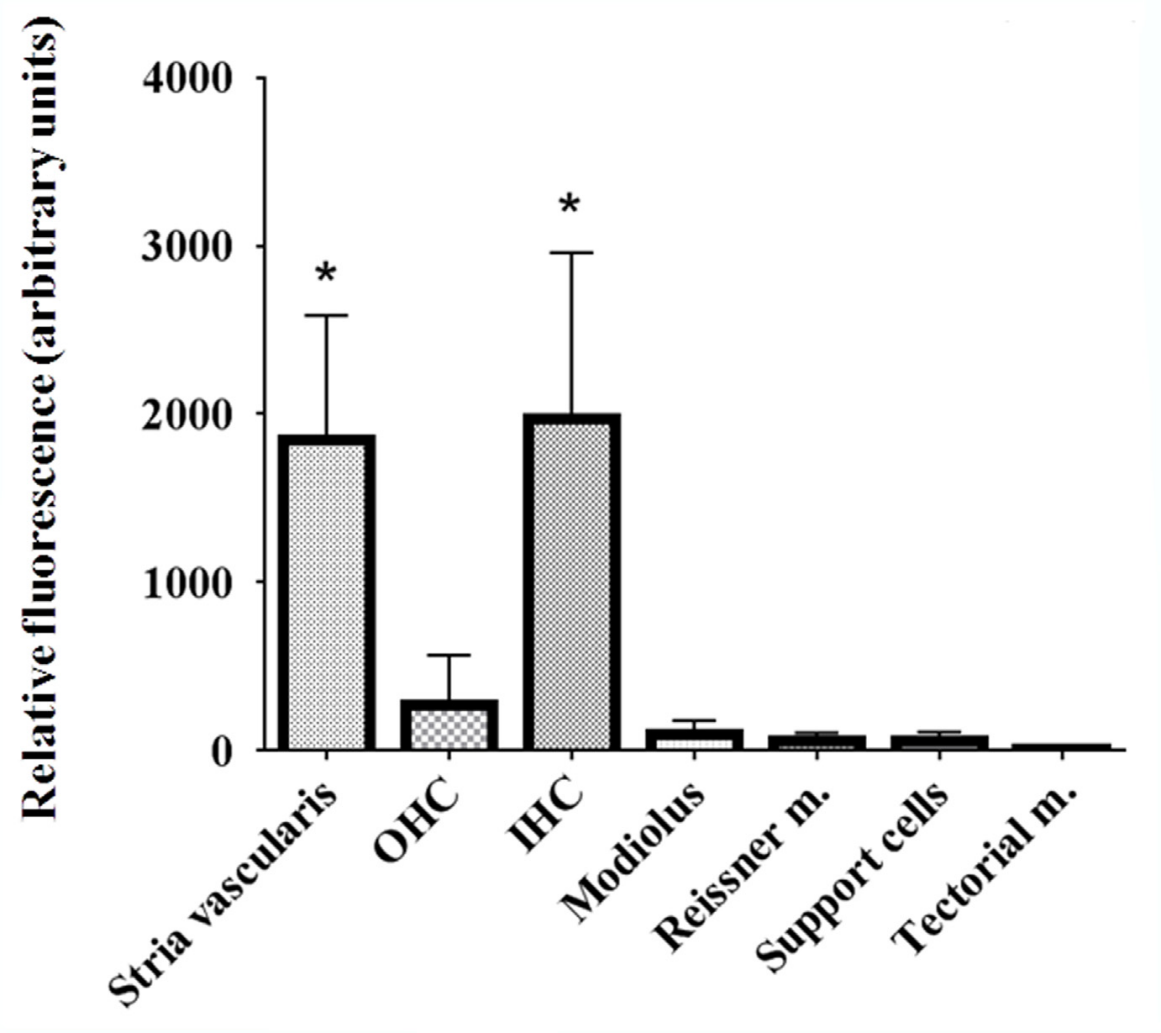
Relative quantification of CB_2_ immunofluorescence labeling intensity in different structures of the cochlea by ImageJ. Support cells: supporting cells (including Deiters’, Hensen’s, Claudius and pillar cells). Tectorial m: tectorial membrane (acellular). Reissner m.: Reissner membrane. OHC: outer hair cells. IHC: inner hair cells. Relative immunofluorescence in the IHC and in the stria vascularis was significantly than in the other cell types. The diagrams include the mean, the standard deviation (n = 10), and the ANOVA results (difference statistically significant respect tectorial membrane *p<0.05).

**Fig 7:**
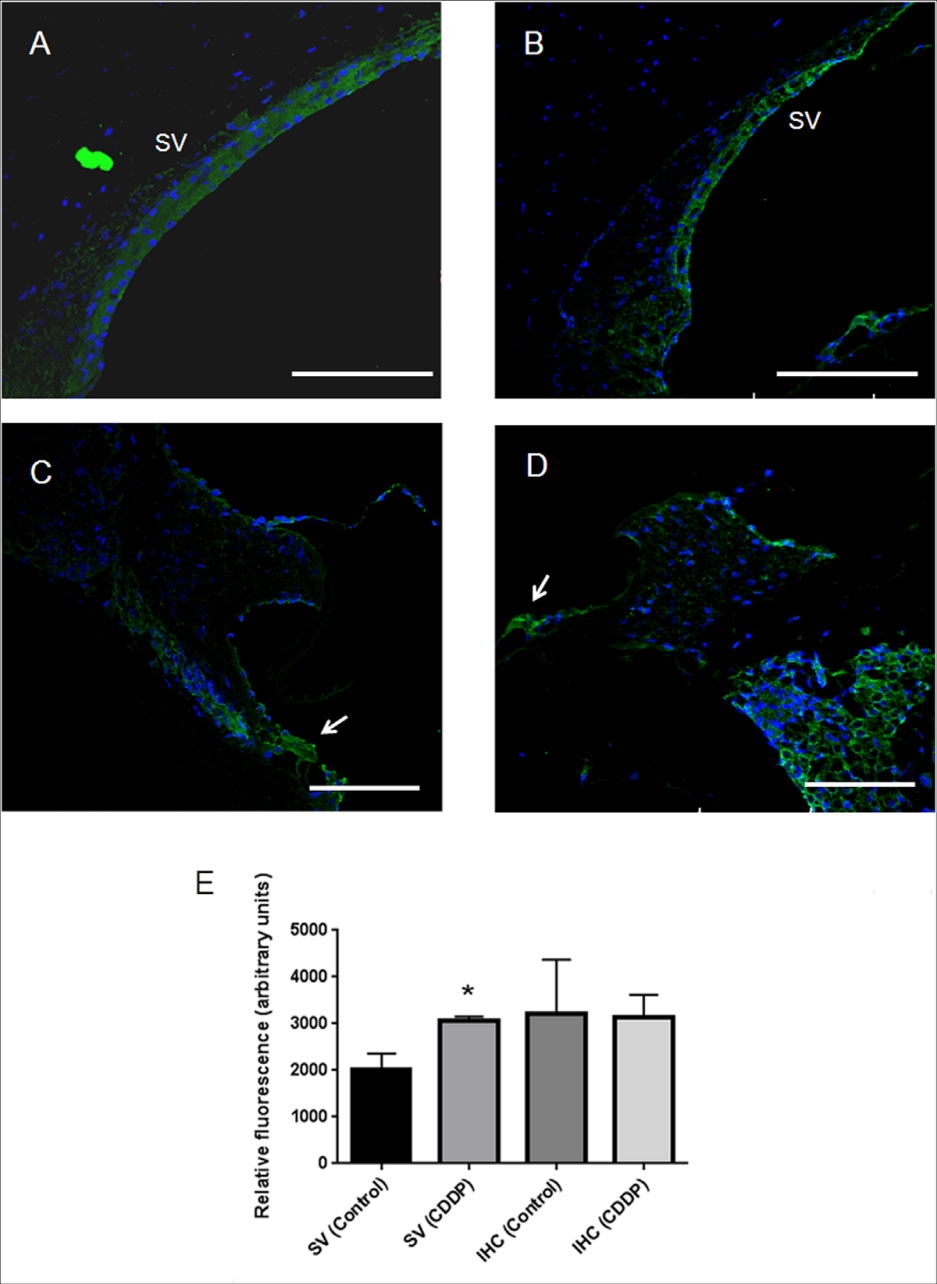
Relative quantification of CB_2_ immunofluorescence labeling intensity in healthy and CDDP-treated animals by ImageJ. Detailed view of the stria vascularis (SV) and the IHC (arrowhead) of healthy (A and C) and CDDP-treated animals (B and D) (Scale bar = 100μm). Relative immunofluorescence in the IHC and in the stria vascularis of healthy and CDDP-treated animals (E). CB_2_ relative immunofluorescence was significantly higher only in the stria vascularis of animals treated with CDDP if compared with the control group. The diagram include the mean, the standard deviation (n = 6; 3 control and 3 CDDP-treated), and the ANOVA results (difference statistically significant in each cellular structure between healthy and CDDP-treated animals *p<0.05).

Relative quantification of CB_2_ immunofluorescence labeling intensity in the IHC and OHC, the stria vascularis, modiolus, Reissner and tectorial membranes and the supporting cells of the rat inner ear is presented in **Fig 6**. OHC as well as supporting cells were almost completely devoid of CB_2_ staining. Relative immunofluorescence of OHC was remarkably lower when compared to labeling of IHC and was not statistically significant (**Fig 6**). The same protocol was assayed to compare relative fluorescence in in the stria vascularis (**Fig 7A and 7B**) and IHC (**Fig 7C and 7D**) of healthy and CDDP-treated animals. CB_2_ immunofluoresce was higher in the stria vascularis of CDDP-treated animals in a statistically significant way (**Fig 7E**). No difference between IHC of healthy and CDDP-treated animals was achieved.

### CB_2_ gene expression in the cochlea and CDDP induced an up-regulation in their expression

**Fig 8:**
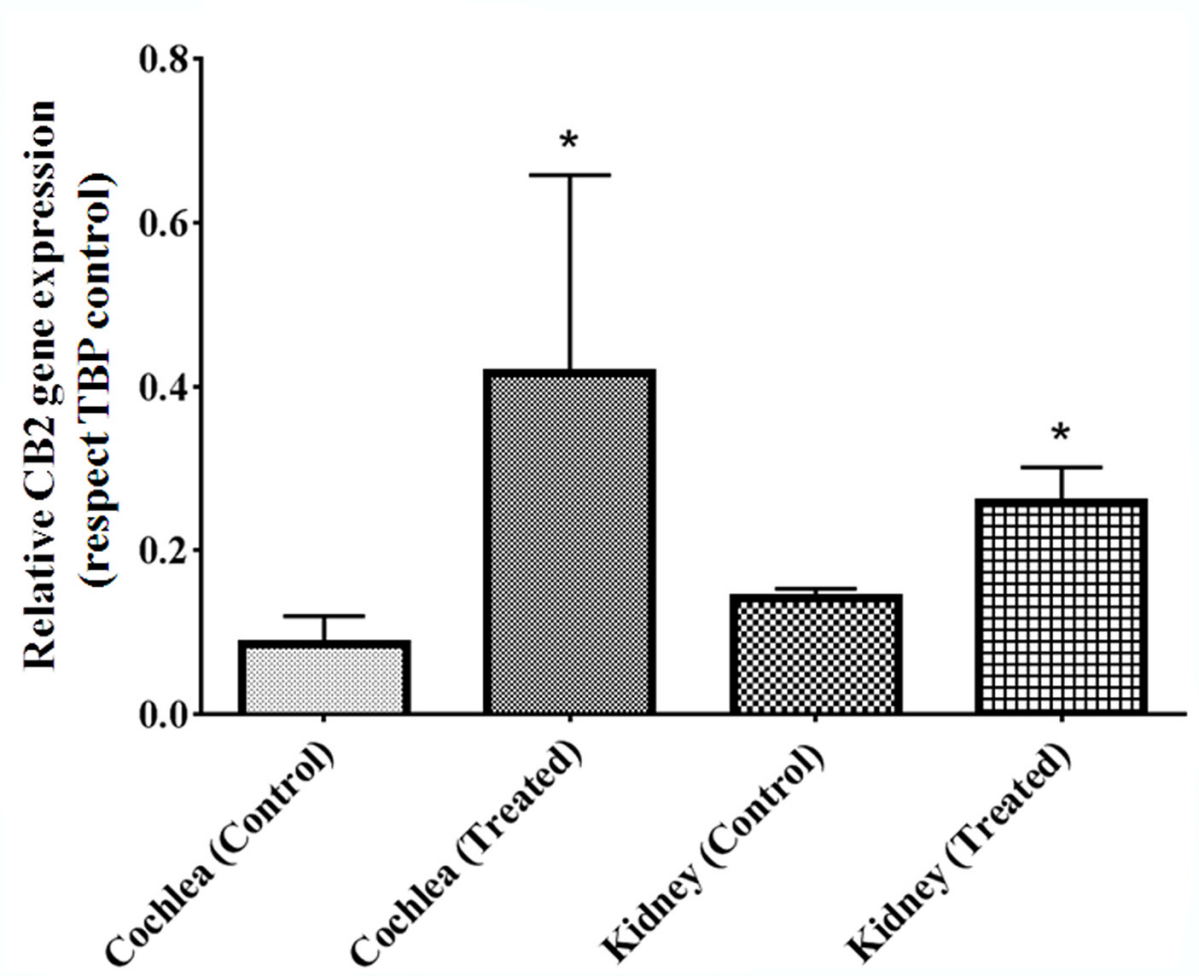
Measure of relative CB_2_ gene expression by RT-qPCR. Measure of CB_2_ gene expression in the cochlea and kidney of healthy (control) and CDDP treated animals, respect TBP reference. CB_2_ gene expression was significantly higher in animals treated with CDDP than those in control group. The diagrams include the mean, the standard deviation (n = 10), and the ANOVA results (difference statistically significant respect control *p<0.05).

Gene expression of CB_2_ receptors was studied in the cochlea of healthy animals with respect to TBP reference gene. The relative levels of expression showed up-transcriptional regulation of CB_2_ under CDDP treatment in a statistically significant way. Similar results were observed in kidney of animals untreated, an under CDDP treatment (**Fig 8**).

Level of CB_1_ gene expression in both organs was also measured as a control. CB_1_ receptors expression was detected in the cochlea and in the kidney. A slightly down-transcriptional regulation in the CDDP treated animals was observed in the cochlea, but there was no statistically significant difference between control and CDDP-treated groups (**see S2 Fig)**.

## Discussion

The expression of cannabinoid receptors in a specific tissue opens the door to potential treatments with agonists and antagonists that could trigger certain cell signaling pathways. Expression of CB_2_ has also been described in astrocytes and some neuron subpopulations. Role of CB_2_ receptor in the central nervous system is related to neuroinflammation and neurodegeneration [31]. In the peripheral nervous system, CB_2_ agonists have analgesic effects by acting at dorsal root ganglia (as well as at spinal cord) [32]. Other authors have reported CB_2_ expression on human skin nerve fibers [33]. Baek *et al*. previously described expression of CB_2_ protein receptor in the vestibular and cochlear nuclei of the brain in a rat model, suggesting that it could have an important role in the control of balance and hearing function [34]. As part of the peripheral nervous system [35], it is not surprising to find such a remarkable immunolabelling of CB_2_ receptor in organ of Corti cells, although its functional role has still to be ascertained. Immunolabeling to CB_2_ receptor resulted to be higher in the stria vascular of animals which received the chemotherapeutic, but no difference could be appreciate in the IHC of healthy and CDDP-treated animals. Due to these limitations of immunohistochemistry to appreciate differences in the transcriptional up-regulation of CB_2_ between healthy and CDDP-treated animals, which suffered big structural tissue degradation, RT-qPCR was assayed to determine the regulation of CB_2_ gene expression.

In 2005 meeting of the Association for Research in Otolaryngology (ARO), Fauser *et al*. reported preliminary studies of the expression of CB_1_ cannabinoid receptor in type I and type II spiral ganglion cells that was higher after intratympanic treatment with salicilate and glutamate in comparison with saline, with no specific stain in the organ of Corti or stria vascularis [36]. In this study high values of CB_1_ gene expression was observed and used as a control of cannabinoid gene expression **(see S2 Fig)**, probably due to the presence of the spiral ganglion in the dissected cochleae. A slightly down-regulation of CB_1_ gene expression in the cochlea and in the kidney was observed when animals received CDDP treatment, being statistically significant in the kidney probably due to a primary down-regulation trying to ameliorate tissue damage promoted by CB_1_ in nephropathy models [37]. Recent studies go deeper in the role of CB_1_ receptor in hearing function. Zheng *et al*. use CBD and Δ-9-THC, and concluded that their results suggest that cannabinoids, such as Δ-9-THC and CBD, may actually aggravate tinnitus, probably because of the net effect of activation of CB_1_ receptors in the dorsal cochlear nucleus might be to increase the excitation of fusiform cells, thus exacerbating neuronal hyperactivity [38]. In a study published in Hearing Research, researchers showed in knockout CB_1_ mice, the role of the cannabinoid receptor in hearing. Animals without CB_1_ presents deficit in their audiograms for frequencies above 8 kHz, but they are still able to distinguished changes in frequencies as well as the wild type [39].

Up to our knowledge, this is the first *in vivo* report of the expression of CB_2_ receptors in the inner ear of mammals. Expression of CB_2_ gene had been previously described in the *in vitro* line HEI-OC1, by means of RT-qPCR [25]. The authors demonstrated that JWH-015 (a synthetic CB_2_ agonist) and HU210 (CB_1_ agonist), could inhibit CDDP-induced apoptosis in HEI-OC1 cells. Specifically, they found that JWH-015 inhibited CDDP-induced caspase-3 and caspase-8 activity; cytochrome c (proapoptotic molecule) release from the mitochondria; increased phosphorylation of ERK; blocked the increase of ROS produced by CDDP; and inhibited TNF-α production on HEI-OC1 cells. From this, they suggested that CB activation was important in CDDP-induced apoptosis of HEI-OC1 cells.

HEI-OC1 cells express several molecular markers characteristic of organ of Corti sensory cells and could be considered as precursors. CB_2_ immunolabeling in our study was seen in inner hair cells, but not in outer hair cells. Previous studies suggested that hair cell types derived from different progenitor cells. Specifically, these studies suggest that IHC derive from the greater epithelial ridge and the OHC derive from the lesser epithelial ridge [40, 41]. This difference during ontogenesis has been related to the lateral process of inhibition that avoid cells from follow the same developmental pathway [42]. These differences in the development of IHC and OHC could explain the differential immunolabeling of CB_2_ receptor in sensory cells reported in our assays.

Our study suggests the expression of CB_2_ receptor mostly in the intermediate region of the stria vascularis, although luminal and basal labeling could also be seen. We consider this as a limitation of our study and further studies would be needed to ascertain what cellular types are specifically expressing CB_2_ receptor. Potential labeling of residual erythrocytes within the capillary vessels of stria vascularis was contemplated, but we consider that it small size is negligible in relation to strial cells. The fact that CB_2_ immunolabeling is also located in the stria vascularis suggests that CB_2_ receptors might play a role in generation and/or maintenance of endocochlear potential. Potential interaction with structures identified in stria vascularis that are related to water and ion transport, antioxidant defenses and other homeostatic mechanisms merit further research.

CDDP clinical use is limited due to the induced toxicity affecting nervous system, kidney function and hearing. CDDP induces apoptosis by binding to DNA, ROS accumulation, increased lipid peroxidation and Ca^2+^ influx and inflammation events [43]. In the present study CDDP treated animals presents an up-regulation in the expression of CB_2_ gene in cochleae and also in the kidney. Mukhopaday *et al*. used an agonist of CB_2_ receptor and CB_2_ knockout mice trying to understand the role of the endocannabinoid system in CDDP-induced kidney disease. They observed that treatment with CB_2_ agonist HU-308 ameliorates many CDDP-induced events like: ROS and inflammation markers; inflammatory cell infiltration and chemokine production; leading to a marker improvement of the renal function in CDDP-treated animals that also receive HU-308. An increase in inflammation, oxidative stress and cell death in knockout mice CB_2_ -/- when compared to wild type was also observed, suggesting the protective role of the CB_2_ receptor activation [44]. More recently, Mukhopaday *et al*. characterized the partial CB_2_ receptor agonist LEI-101 showing promising results. The partial agonist does not induce cannabimimetic in animals treated, and a considerable attenuation in CDDP-induced nephropathy, including inflammation and oxidative stress [45].

As shown in this study, CDDP treatment increased the gene expression levels of CB_2_ in the cochlea and in kidney (control) (**Fig 8**). This up-regulation of the inflammatory microenvironment induced by the treatment with CDDP promotes the overexpression of endocannabinoid system (increased CB_2_). CB_2_ overexpression may represent an adaptive response to control a deregulated balance in the ear maybe trying to restore normal conditions. A deeper understanding of the functional role of CB_2_ receptor in sensory and non-sensory elements of the cochlea may help develop therapies using CB_2_ as target for reducing damage to the hearing organ. That opens a big field of possibilities to the treatment of inner ear diseases which involves inflammation process, using drugs with non canabimimetic effects, because CB_2_ is the lack of psychoactive side effects after stimulation.

## Conclusions

In conclusion, evidence for the presence of cannabinoid CB_2_ receptor by immunohystochemistry and by RT-qPCR was provided. An immunolabeling of CB_2_ antibodies in four structures of the adult rat cochlea was found. That was, stria vascularis, inner hair cells, auditory afferent nerves and cell bodies of the spiral ganglion. Up-regulation of CB_2_ gene expression in animals exposed to CDDP treatment was also detected, when compared with healthy animals. This fact was partially supported by the higher immunofluorescence observed in the stria vascularis of CDDP-treated animals if compared with the healthy ones. These results suggest a considerable promising potential of CB_2_ receptor as a target of new treatments against CDDP-induced ototoxicity, and probably against other inflammatory diseases in the inner ear. Further research is needed to determine the functionality of CB_2_ receptors in the organ of Corti and the potential therapeutic role of agonists and antagonists of these receptors.

## Supporting Information

S1 Fig Detailed view of tissue sections pre adsorbed with CB_2_ blocking peptide. Detailed view of the cochlea pre adsoberd with CB_2_ blocking peptide. No immunofluorescence was observed in the stria vascularis (SV), the IHC or the spiral ganglion (SG) (Scale bar = 100μm).
(TIF)

S2 Fig Measure of relative CB_1_ gene expression by RT-qPCR. Measure of relative CB_1_ gene expression in the cochlea and kidney of healthy (control) and CDDP treated animals, respect TBP reference. The diagrams include the mean, the standard deviation (n = 10), and the ANOVA results (difference statistically significant respect control *p<0.05).
(TIF)
