# Supplementary figures and images for: Spontaneous Cannabinoid Receptor 2 (CB2) Expression in the Cochlea of Adult Albino Rat and Its Up-Regulation after Cisplatin Treatment
Source: PLoS One. 2016 Aug 26;11(8):e0161954. doi: 10.1371/journal.pone.0161954 (PMC5001640; doi:10.1371/journal.pone.0161954)

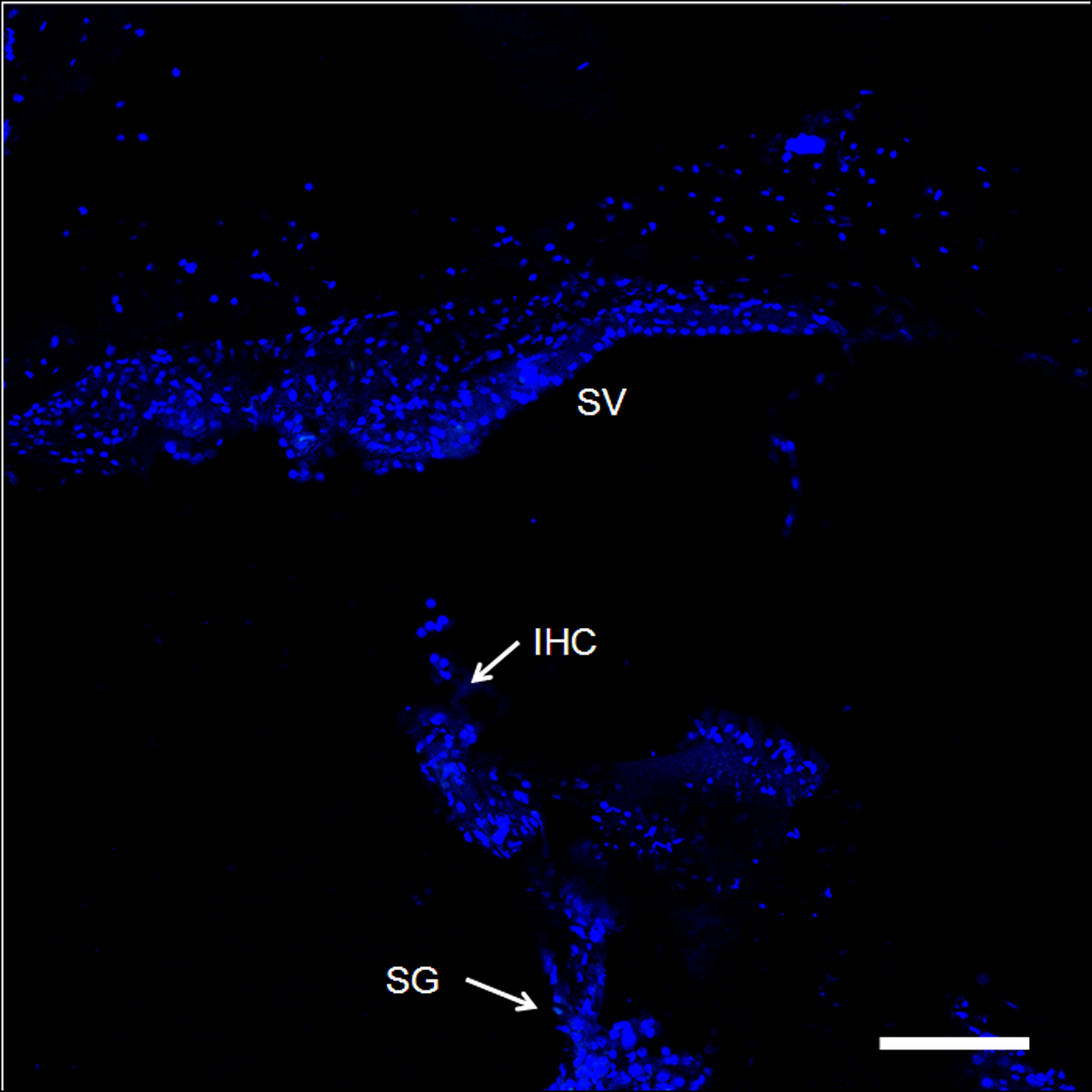

Supplement: S1 Fig — Detailed view of the cochlea pre adsoberd with CB2 blocking peptide. No immunofluorescence was observed in the stria vascularis (SV), the IHC or the spiral ganglion (SG) (Scale bar = 100μm). (TIF) [file pone.0161954.s001.tif]

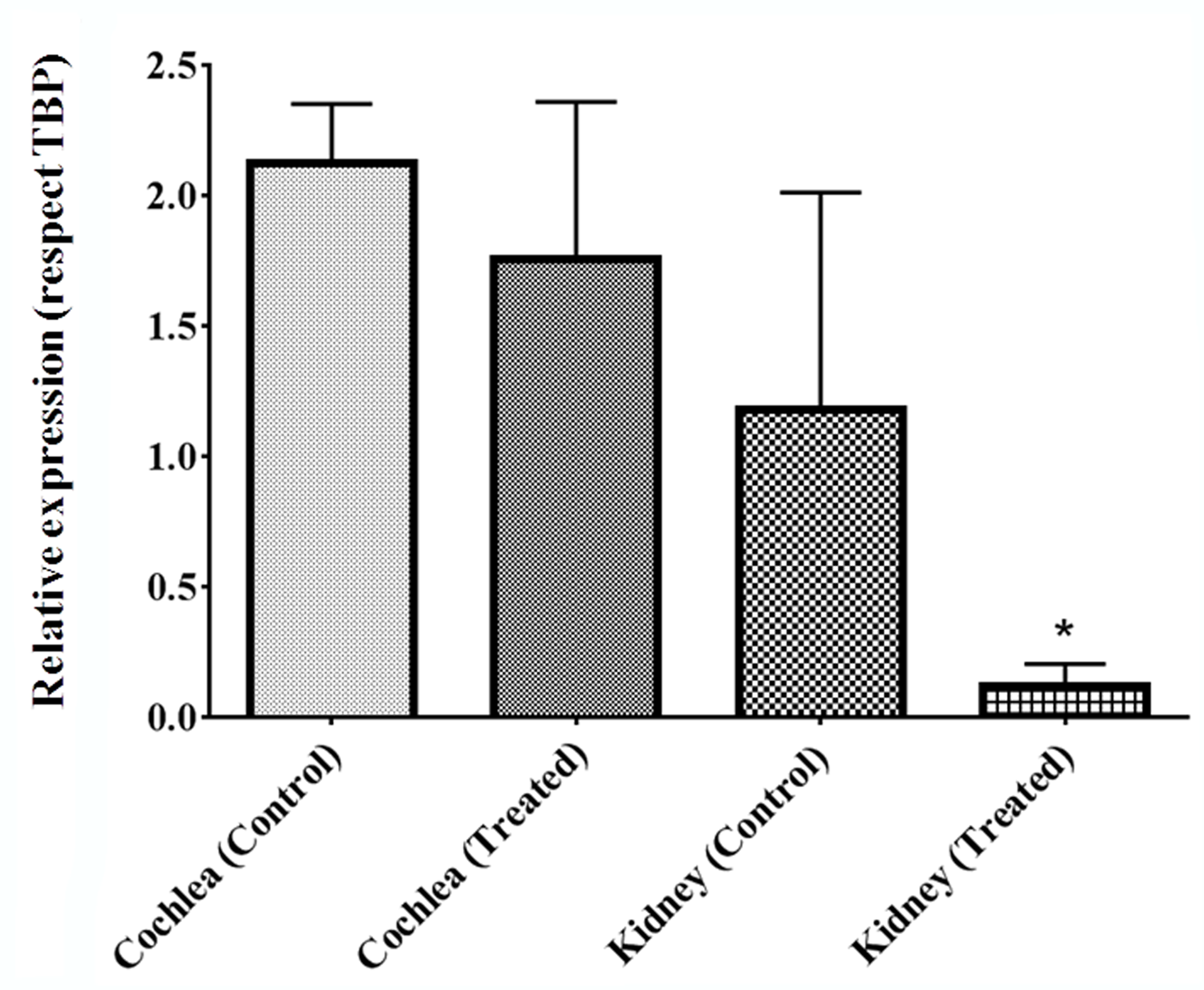

Supplement: S2 Fig — Measure of relative CB1 gene expression in the cochlea and kidney of healthy (control) and CDDP treated animals, respect TBP reference. The diagrams include the mean, the standard deviation (n = 10), and the ANOVA results (difference statistically significant respect control *p<0.05). (TIF) [file pone.0161954.s002.tif]
